# Overexpression of *Hdac6* enhances resistance to virus infection in embryonic stem cells and in mice

**DOI:** 10.1007/s13238-014-0120-6

**Published:** 2014-12-09

**Authors:** Dekun Wang, Qingwen Meng, Lihong Huo, Meng Yang, Lingling Wang, Xinyu Chen, Jianchao Wang, Zhiguo Li, Xiaoying Ye, Na Liu, Qiuyan Li, Zhen Dai, Hongsheng Ouyang, Ning Li, Jun Zhou, Lingyi Chen, Lin Liu

**Affiliations:** 1 State Key Laboratory of Medicinal Chemical Biology, Collaborative Innovation Center for Biotherapy, Department of Genetics and Cell Biology, College of Life Sciences, Nankai University, Tianjin, 300071 China; 2 State Key Laboratory of Veterinary Biotechnology, Harbin Veterinary Research Institute, Chinese Academy of Agricultural Sciences, Harbin, 150001 China; 3 State Key Laboratory for Agrobiotechnology, College of Biological Sciences, China Agricultural University, Beijing, 100193 China; 4 College of Animal Science and Veterinary Medicine, Jilin University, Changchun, 130062 China

**Dear Editor**,

Histone deacetylase 6 (*Hdac6*) is a mostly cytoplasmic class II HDAC. Many proteins have been identified as substrates of Hdac6. Among them, the most well characterized substrate of Hdac6 is α-tubulin. Through deacetylating acetylated lysine 40 in α-tubulin, Hdac6 modulates the acetylation of microtubules (Hubbert et al., 2002).

Increasing evidences suggest that infection of various types of viruses, including HIV and influenza A virus, is associated with upregulated acetylation level of tubulin or Tat in cultured cells. Hdac6 activity is downregulated in infected cells, consequently resulting in elevated levels of acetylated tubulin or Tat (Huo et al., 2011; Valenzuela-Fernandez et al., 2005). Consistently, overexpression of active *Hdac6* inhibits the acetylation of α-tubulin, and remarkably, prevents HIV-1 envelope-dependent cell fusion and infection, without affecting the expression and co-distribution of HIV-1 receptors (Valenzuela-Fernandez et al., 2005). In contrast, knockdown of *Hdac6* or inhibition of its tubulin deacetylase activity strongly enhances HIV-1 infection and syncytia formation (Valenzuela-Fernandez et al., 2005). Virus replication is also enhanced in Hdac6-depleted cells, demonstrating that Hdac6 is an essential component of innate antiviral immunity (Nusinzon and Horvath, 2006). However, it remains to be determined whether Hdac6 plays a role in anti-virus infection in a whole animal model.

To test the anti-virus effect of Hdac6 in an animal model, we first constructed a *Hdac6* transgenic (*Hdac6*^*tg*^) embryonic stem (ES) cell line. An overexpression vector, containing the *Hdac6*-IRES-*Puro* cassette downstream of the chicken *β*-*Actin* (CAG) promoter, was used to construct the *Hdac6*^*tg*^ ES cells (Fig. S1A). The integration and the expression of *Hdac6* transgene were verified by genomic DNA PCR and RT-PCR (Fig. S1B–D). Levels of Hdac6 protein also were remarkably higher in *Hdac6*^*tg*^ ES clones than in WT ES controls by Western blot analysis (Fig. S1E and S1F). Consistently, the level of tubulin acetylation was reduced in *Hdac6*^*tg*^ ES clones, using β-actin as loading control.

We previously demonstrated efficient generation of transgenic mice by the method of injection of ES cells into 4–8-cell embryos (Huang et al., 2008). Prior to making transgenic mice, we tested whether ES clones overexpressing *Hdac6* show resistance to adenovirus infection. A recombinant human adenovirus type 5 (dE1/E3) expressing GFP (Ad-GFP) was used to infect WT and *Hdac6*^*tg*^ ES clones, so that the infection of adenovirus can be indicated by the expression of GFP. Adenovirus infection does not affect the growth of WT and *Hdac6*^*tg*^ ES cell. Rather, *Hdac6*^*tg*^ ES clones showed reduced number of infected cells, compared to control ES clones at 24, 36, and 48 h after infection (Fig. 1A and 1B). Also, the adenovirus titers affected the efficiency of infection. At lower titers e.g. Ad-GFP virus stock at 1 × 10^6^ ifu/mL, similar fractions of *Hdac6*^*tg*^ ES cell and control WT clones were infected by adenovirus, as indicated by GFP positivity, at 36 h after infection (Fig. 1C and 1D). However, as virus titers increased to 1 × 10^7^ or 1 × 10^8^ ifu/mL, less *Hdac6*^*tg*^ ES cells, particularly ES cell clone #43, were infected by adenovirus, compared to the control WT ES cell clones. Evident resistance to higher titer (10^8^ ifu/mL of Ad-GFP) but similar resistance to lower titer (10^6^ ifu/mL of Ad-GFP) of adenovirus infection also were found in another independent *Hdac6*^*tg*^ ES cell clone 9# by flow cytometry, in comparison with control WT BF10 ES cell clones, at 36 h after infection (Fig. 1E and 1F). These data suggest that ES cells overexpressing *Hdac6* display resistance to infection by adenovirus at high titers.

**Figure 1:**
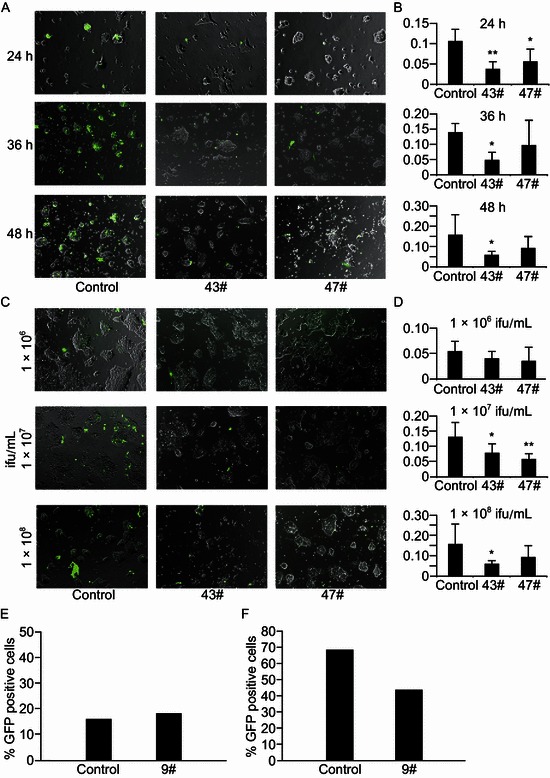
**ES cells overexpressing*Hdac6*show resistance to adenovirus infection**. (A) Representative images showing infection of GFP-expressing adenovirus (Ad-GFP, 1 × 10^8^ ifu/mL) in *Hdac6*^*tg*^ V6.5 ES cell clones compared to control V6.5 ES cell clones, at 24, 36 and 48 h after infection. (B) The proportion of GFP-positive cells out of a total of 300 cells in (A) was analyzed under a fluorescence microscope. Infection frequencies, referring to the percentage of GFP-positive cells, at various hours after infection, were plotted. (C) Representative images showing infection of various titers of Ad-GFP in *Hdac6*^*tg*^ V6.5 ES cell clones compared to control ES clones. (D) Infection frequencies of the cells in (C) were plotted. (E) Wild-type BF10 ES cell clones and *Hdac6*^*tg*^ BF10 ES cell clones were infected with 10^6^ ifu/mL of Ad-GFP. At 36 h after infection, the percentage of GFP-positive cells was measured by flow cytometry. (F) Wild-type BF10 ES clones and *Hdac6*^*tg*^ BF10 ES clones were infected with 10^8^ ifu/mL of Ad-GFP. At 36 h after infection, the percentage of GFP-positive cells was measured by flow cytometry. *P < 0.05, **P < 0.01, compared to controls

Stable ES cell clones overexpressing *Hdac6* were injected into 4–8 cell embryos of albino ICR recipient mice. *Hdac6*^*tg*^ chimera mice were generated (Fig. S2A and S2B). Through germline transmission, *Hdac6*^*tg*^ chimera mice gave birth to F1 mice identified by coat color (Fig. S2C). Genotyping of F1 mice showed that most of F1 mice harbored the *Hdac6* transgene (Fig. S2D). Consistent with genotyping results, *Hdac6*^*tg*^ F1 mice showed elevated expression levels of *Hdac6* measured by quantitative RT-PCR, in contrast to minimal *Hdac6* expression of non-transgenic mice (#4 and #9) (Fig. S2E). Thus far, we have obtained more than 100 F1 and F2 mice. *Hdac6* transgenic mice exhibited normal fertility and sex ratio. All 128 *Hdac6*^*tg*^ mice are generally healthy, with the exception of only one mouse showing abnormal neck growth.

Next, we tested whether the *Hdac6*^*tg*^ mice are resistant to virus infection. Infected with avian H5N1 virus, WT mice died one day earlier than did *Hdac6* transgenic mice (Fig. 2A). While WT mice had survival rate of 85% (6/7) on day 3, all *Hdac6* transgenic mice survived. Moreover, only 30% of WT mice survived by the end of experiments day 12, while 70% of *Hdac6* transgenic mice were still alive on day 12. Survival rate of 11-week-old *Hdac6* transgenic mice also was increased. In addition, the body weight of 5-week-old WT mice reduced significantly 7 days after infection, whereas age-matched *Hdac6* transgenic mice maintained their body weight stably (Fig. 2B). Body weight of 11-week-old WT mice was also reduced 7 days after virus infection. In contrast, age-matched *Hdac6* transgenic mice maintained body weight by the end of experiment day 12 (Fig. 2C). Statistical analysis revealed that the body weight did not differ significantly between the surviving *Hdac6*^*tg*^ mice and WT mice, likely due to the small number of mice used and loss of dead mice for comparison. Actually, only one 5-week-old WT mouse and one 7-week-old WT mouse survived 10 days and 7 days after virus infection, respectively. Expression levels of *Hdac6* in the transgenic mice, regardless of death or live, were generally higher than those in WT and ICR mouse controls (Fig. 2D and 2E). However, no correlation between the expression levels of *Hdac6* and the survival of mice was observed in *Hdac6*^*tg*^ mice. These data suggested that pro-survival effect of *Hdac6* overexpression is dose-independent once its expression level exceeds a threshold.

**Figure 2:**
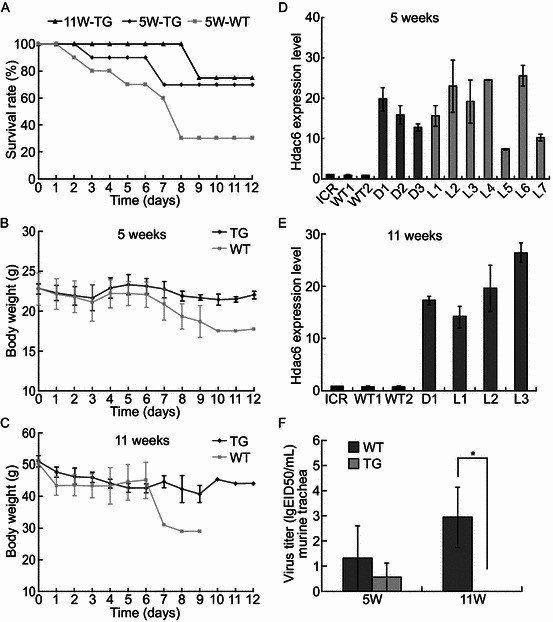
**Survival rate and body weight of Hdac6 transgenic mice after virus infection**. (A) Survival rate of Hdac6 transgenic mice at 5-week (*n* = 10) or 11-week-old (*n* = 6) and wide-type mice (*n* = 10) at 5-week-old. Lethality of avian H5N1 influenza virus was compared for TG and WT mice. Groups of 10 mice were infected i.n. with 0.8 LD50 virus and examined daily for 12 days. (B) Body weight of Hdac6 transgenic and wide-type mice at the age of 5 weeks. Mean body weight variation is compared among different TG and WT mice infected i.n. with 0.8 LD50 or 1.1 LD50 virus and examined daily for 12 days. (C) Body weight of Hdac6 transgenic and wide type mice at the age of 11 weeks. Some time points for 11-week-old mice were missing due to collection for other analysis, including virus replication. (D and E) Relative expression levels of *Hdac6* in *Hdac6* transgenic mice, compared with WT and ICR controls at 5-week (D) or 11-week-old (E). D, dead; L, live. (F) Mice were inoculated with 0.8 or 1.1 LD_50_ of virus. Trachea were then collected on day 3 and 9 and titrated in embryonated chicken eggs. The mean virus titers (log_10_EID_50_/mL) at two time points from two mice per group are shown (Mean ± S.E., *n* = 4)

Furthermore, the virus titers varied among mice infected with the virus. The virus titers in the trachea of *Hdac6* transgenic mice at the age of 11 weeks were significantly reduced compared to control (*P* < 0.05) (Fig. 2F).

High pathogenic avian H5N1 influenza A viruses occasionally infect humans, and a most recent study shows that a reassortant H5 HA/H1N1 virus—comprising H5 HA (from an H5N1 virus) with four mutations is capable of viral transmission in mammals (Imai et al., 2012). We show that mice are readily susceptible to avian H5N1 influenza virus infection, and that mice with overexpression of *Hdac6* show enhanced resistance to H5N1 virus, as demonstrated by postponed death, reduced death rate, and body weight maintenance. We anticipate that these initial findings will likely be substantiated by a large-scale experiment with various types of viruses.

We speculate that the increased anti-virus capacity of ES cells and mice might employ similar mechanisms of suppression of virus infection shown in other cell types. The plasma membrane is the first site where viruses enter the cells. The cytoskeletal components underlying plasma membrane including microtubules and actin are involved in virus entry into host cells. Several viruses, such as HIV-1 and influenza A virus, induce acetylation of tubulin to enable efficient infection and spreading (Husain and Harrod, 2011; Valenzuela-Fernandez et al., 2005). Hdac6 is a cytoplasmic deacetylase associated with cytoskeleton that uniquely mediates deacetylation of α-tubulin and cortactin, and promotes cell motility (Hubbert et al., 2002; Zhang et al., 2007). Deacetylation of α-tubulin by increased expression of *Hdac6* reduces fusion of viruses with plasma membrane and enhances resistance to virus entry, while reduced or inhibition of *Hdac6* increases acetylated tubulin and facilitates virus-cell fusion and infection (Valenzuela-Fernandez et al., 2005). The deacetylase activity of Hdac6 on tubulin also links to immune synapse organization (Serrador et al., 2004). Moreover, autophagy may protect against virus infection through recognizing signatures of virus infection, degradation of viral components (xenophagy), and restriction of virus replication (Lee and Iwasaki, 2008), and Hdac6 promotes autophagy and stimulates autophagosome-lysosome fusion and substrate degradation (Lee et al., 2010).

Concerns still exist about potential risks of *Hdac6* overexpression in tumorigenesis. *HDAC6* mRNA appears to express at higher levels in some cancers, including breast cancer and oral squamous cell carcinoma (Sakuma et al., 2006; Zhang et al., 2004). Fibroblasts deficient in *Hdac6* are more resistant to both oncogenic Ras and ErbB2-dependent transformation, and *Hdac6*-null mice are more resistant to chemical carcinogen-induced skin tumors (Lee et al., 2008). Cell culture *in vitro* shows that expression of *Hdac6* and deacetylated tubulin are associated with tumorigenesis, cellular motility and cancer cell migration and invasion (Rey et al., 2011). Yet, whether high expression of *Hdac6* leads to tumorigenesis *in vivo* remains unclear. We found that *Hdac6* transgenic mice are healthy and actually show high reproductive performance. These mice still produce litter size of 12 on average by the age of 7–10 months, like normal mice of the same genetic background at younger age (2–3 months). From 128 *Hdac6* transgenic mice we obtained thus far, only one female exhibited abnormal growth of the neck by the age of 10 months. Thus, mice with overexpression of *Hdac6* do not show noticeably increased tumorigenesis. Yet, more extensive studies are required to follow those mice regarding long-term effects of Hdac6 overexpression. We are undertaking experiments by continuous monitoring the health conditions. The *Hdac6*^*tg*^ mice reported in this study provide the proof of principle of anti-virus function by Hdac6 *in vivo*. In future, site-specific targeted transgenic mice would be more informative to further validate the function of *Hdac6* in anti-virus studies.

## Footnotes

We thank Drs. Xiaohong Zhang, Zhonghua Liu, and Wentao Qiao for help and discussion. This work was supported by the Ministry of Agriculture of China Transgenic Special Program (2009ZX08006-010B, 2009ZX08006-001B), the National Basic Research Program (973 Program) (Nos. 2011CBA01002 and 2009CB941000), the National Science and Technology Major Project of China (2012ZX10001-006), and the Natural Science Foundation of Tianjin, China (No. 14JCYBJC23600).

Dekun Wang, Qingwen Meng, Lihong Huo, Meng Yang, Lingling Wang, Xinyu Chen, Jianchao Wang, Zhiguo Li, Xiaoying Ye, Na Liu, Qiuyan Li, Zhen Dai, Hongsheng Ouyang, Ning Li, Jun Zhou, Lingyi Chen, and Lin Liu declare no conflict of interest.

All institutional and national guidelines for the care and use of laboratory animals were followed.

## Electronic supplementary material

Below is the link to the electronic supplementary material.

Supplementary material 1 (TIFF 738 kb)

Supplementary material 2 (TIFF 1683 kb)
