# Supplementary figures and images for: Overexpression of Hdac6 enhances resistance to virus infection in embryonic stem cells and in mice
Source: Protein Cell. 2014 Dec 9;6(2):152–6. doi: 10.1007/s13238-014-0120-6 (PMC4312767; doi:10.1007/s13238-014-0120-6)

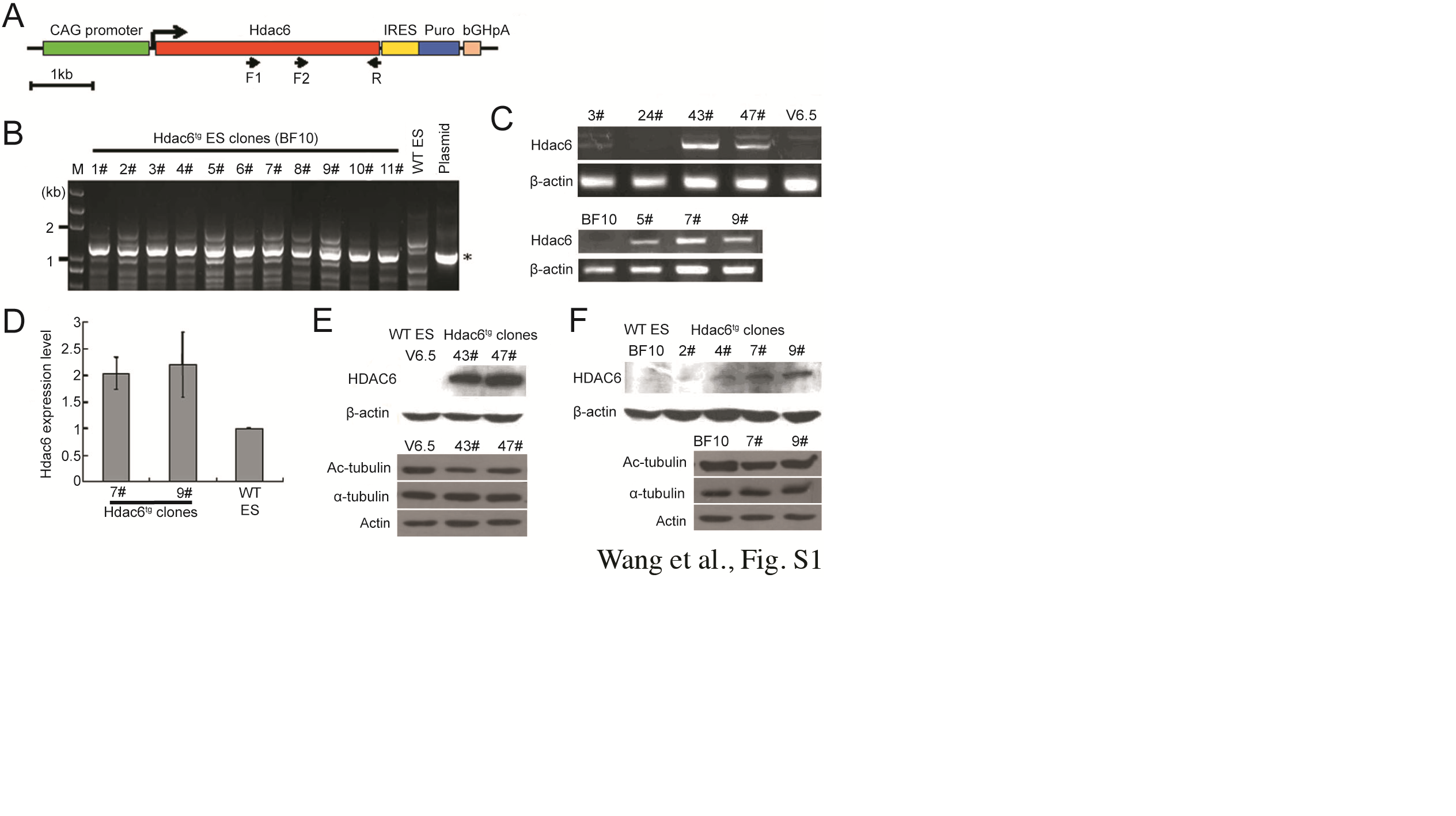

Supplement: Supplementary file 1 — Supplementary material 1 (TIFF 738 kb) [file 13238_2014_120_MOESM1_ESM.tif]

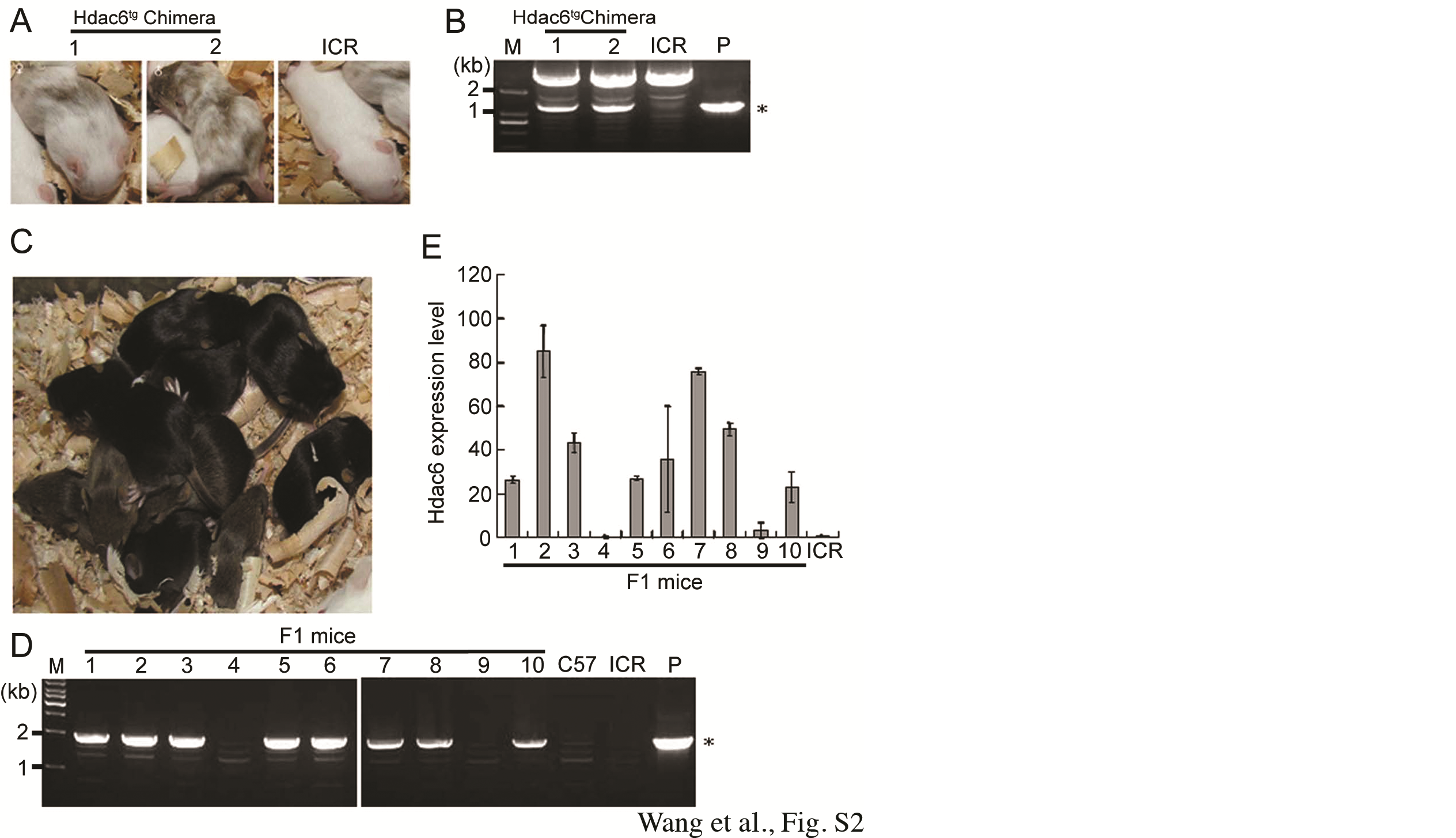

Supplement: Supplementary file 2 — Supplementary material 2 (TIFF 1683 kb) [file 13238_2014_120_MOESM2_ESM.tif]
